# The 23-valent pneumococcal polysaccharide vaccine in patients with rheumatoid arthritis: a double-blinded, randomized, placebo-controlled trial

**DOI:** 10.1186/s13075-016-1207-7

**Published:** 2017-01-25

**Authors:** Yasumori Izumi, Manabu Akazawa, Yukihiro Akeda, Shigeto Tohma, Fuminori Hirano, Haruko Ideguchi, Ryutaro Matsumura, Tomoya Miyamura, Shunsuke Mori, Takahiro Fukui, Nozomi Iwanaga, Yuka Jiuchi, Hideko Kozuru, Hiroshi Tsutani, Kouichirou Saisyo, Takao Sugiyama, Yasuo Suenaga, Yasumasa Okada, Masao Katayama, Kenji Ichikawa, Hiroshi Furukawa, Kenji Kawakami, Kazunori Oishi, Kiyoshi Migita

**Affiliations:** 1 Japanese National Hospital Organization (NHO) (Evidence-based Medicine Study Group), Higashigaoka 2-5-23, Meguro, Tokyo, 152-8621 Japan; 2 Department of Public Health and Epidemiology, Meiji Pharmaceutical University, Noshio 2-522-1, Kiyose, Tokyo, 204-8588 Japan; 3 Research Institute for Microbial Diseases, Osaka University, Yamadaoka 3-1, Suita, Osaka 565-8563 Japan; 4 Infectious Diseases Surveillance Center, National Institute of Infectious Diseases, Toyama 1-23-1, Shinjuku, Tokyo, 162-8640 Japan; 5 Department of Rheumatology, Fukushima Medical University, Hikarigaoka 1, Fukushima, 960-1295 Japan

**Keywords:** Interstitial lung disease, Pneumococcal polysaccharide vaccine, Pneumonia, Rheumatoid arthritis

## Abstract

**Background:**

Pneumococcal pneumonia is the most frequent form of pneumonia. We herein assessed the effectiveness of the 23-valent pneumococcal polysaccharide vaccine (PPSV23) in the prevention of pneumonia overall in rheumatoid arthritis (RA) patients at risk for infections. We hypothesized that PPSV23 vaccination is superior in preventing pneumococcal pneumonia compared with placebo in RA patients.

**Methods:**

A prospective, multicenter, double-blinded, randomized, placebo-controlled (1:1) trial was conducted across departments of rheumatology in Japanese National Hospital Organization hospitals. RA patients (*n* = 900) who had been treated with biological or immunosuppressive agents were randomly assigned PPSV23 or placebo (sodium chloride). The primary endpoints were the incidences of all-cause pneumonia and pneumococcal pneumonia. The secondary endpoint was death from pneumococcal pneumonia, all-cause pneumonia, or other causes. Cox regression models were used to estimate the risk of pneumonia overall for the placebo group compared with the vaccine group.

**Results:**

Seventeen (3.7%) of 464 patients in the vaccine group and 15 (3.4%) of 436 patients in the placebo group developed pneumonia. There was no difference in the rates of pneumonia between the two study groups. The overall rate of pneumonia was 21.8 per 1000 person-years for patients with RA. The presence of interstitial pneumonia (hazard ratio: 3.601, 95% confidence interval: 1.547–8.380) was associated with an increased risk of pneumonia in RA patients.

**Conclusion:**

PPSV23 does not prevent against pneumonia overall in RA patients at relative risk for infections. Our results also confirm that the presence of interstitial lung disease is associated with pneumonia in Japanese patients with RA.

**Trial registration:**

UMIN-CTR UMIN000009566. Registered 17 December 2012.

## Background

Patients with rheumatoid arthritis (RA) are at increased risk of serious infections. Although a newer class of biologic disease-modifying antirheumatic drugs (bDMARDs) has significantly advanced the treatment of RA, these drugs are associated with an increased risk of several types of infections [1, 2]. Pneumonia is also one of the major causes of mortality in patients with RA [3]. Influenza and pneumococcal infections are two vaccine-preventable infectious diseases that have been associated with high morbidity in patients with RA caused by immunosuppressive treatments [4, 5]. In view of these data highlighting the increased risk of infectious diseases in RA patients receiving immunosuppressive treatments and the awareness and performance of vaccinations, clear recommendations for vaccinations under the use of biological agents is needed.

*Streptococcus pneumoniae* is the most common cause of community-acquired pneumonia [6]. Recent randomized controlled trials (RCTs) demonstrated that the 23-valent pneumococcal polysaccharide vaccine (PPSV23) is effective in the prevention of invasive pneumococcal disease among high-risk older populations [7]. However, the vaccine’s protective efficacy against pneumococcal pneumonia in immunosuppressed people including patients with autoimmune diseases remains unknown [8]. PPSV23 was licensed more than 30 years ago and is recommended as the standard intervention for the older population (>60 years of age) and adults with underlying diseases [9]. PPSV23 is also strongly recommended for patients with autoimmune inflammatory rheumatic diseases [10]. However, data regarding efficacy of pneumococcal vaccines in patients with RA receiving immunotherapy including biological agents are rare and often conflicting. We therefore conducted a prospective, multicenter, double-blinded, randomized, placebo-controlled trial to determine the efficacy of PPSV23 in patients with RA receiving immunosuppressive treatments. Our primary objective was to assess the effectiveness of PPSV23 in the prevention of pneumococcal pneumonia and pneumonia overall in RA patients at risk of pneumonia.

## Methods

### Study design and patient population

We performed a double-blinded, randomized, placebo-controlled trial. Patients with clinically diagnosed RA were recruited in National Hospital Organization (NHO) hospitals throughout Japan (the trial was conducted in NHO 32 hospitals) from September 2010 to December 2012 [11]. The risk of infections was reported to be associated with their comorbidity and treatments in RA patients [12]. Eligible patients were therefore divided into the following groups: patients with rheumatoid lung disease (*n* = 144), patients treated with biological agents (*n* = 510; TNF inhibitors = 336, toclizumab = 124, abatacept = 50), patients treated with immunosuppressive agents (*n* = 144), patients receiving more than 5 mg/day of prednisone (*n* = 241), and patients classified as Steinbrocker stage 3 or 4 (*n* = 485). Almost all patients (>93%) initiated biologics >3 months prior to study entry. Patients were excluded if they had previously received pneumococcal vaccination. Eligible RA patients were assigned randomly to the vaccine group or the placebo group. A statistician who was not on the study team performed the randomization using a random number table and numbered the containers accordingly.

The following parameters were analyzed when the patient was first admitted to the study: swollen joint count, tender joint count, patient global assessment of disease activity, physician global assessment of disease activity, Health Assessment Questionnaire Disability Index score, serum levels of C-reactive protein (CRP), and Disease Activity Score 28-joint assessment with CRP [13]. This study was registered in UMIN-CTR (www.umin.ac.jp/ctr/UMIN-CTR; UMIN000009566).

### Intervention

Investigators in each NHO hospital registered patient information to the NHO’s central data center through an online system. According to the predetermined allocation code book, either vaccine or placebo (1:1) was simply allocated in the order of registration. Patients were randomly assigned to receive either 0.5 ml (25 μg) of PPSV23 (Pneumovax NP; Merck Sharp & Dohme Corp., Tokyo, Japan) or 0.5 ml of placebo (sodium chloride) subcutaneously in the upper arm. The placebo medication was identical in color. The vaccines were prepared in a masked fashion for those who administered it, blinding both the administrator of the vaccine and the patient to the type of vaccine given. Vaccine and placebo were presented in identical, single-dose syringes and needle combinations that were labeled with sequential study numbers only. A statistician who was not on the study team performed the randomization using a random number table and numbered the containers accordingly.

Patients were instructed to record local reactions (e.g., redness, swelling, and tenderness) and systemic reactions (e.g., fever, nausea, and vomiting). Patients were also monitored for 12 months after enrollment to follow the development of pneumonia, including that stemming from pneumococcal disease.

### Outcome measures and definitions

The primary effectiveness endpoint was the prevention of overall pneumonia using an intention-to-treat (ITT) approach. The primary endpoints were pneumonia and pneumococcal pneumonia. Pneumonia was diagnosed by the medical staff of the respiratory unit at the affiliated hospital according to the presence of clinical symptoms and a new infiltrate on chest radiography. Pneumococcal pneumonia was diagnosed from a positive blood, pleural fluid, or sputum culture or from a positive pneumococcal antigen test result using a urine sample. Information on fatal cases was obtained from a provincial registry, which is based on death certificates written by the attending physicians. During the study period, the patient was instructed to contact the local doctor responsible for the study if he or she developed a fever of 38 °C or greater for more than 3 days, with or without respiratory tract symptoms, or if he or she had any other cause to suspect recurrent pneumonia. Chest radiography was performed upon clinical suspicion of pneumonia. If pneumonia was confirmed, blood, sputum, and urine samples were obtained from the patient, if possible, for etiological diagnosis. Blood testing was performed using routine procedures and quantitative sputum cultures were tested using purulent samples. The secondary effectiveness endpoint was all cases of death.

### Statistical analyses

Data are expressed as the mean (standard deviation) and analyzed using the SPSS software program. For univariate analyses, the χ^2^ test or Fisher’s exact test was used for categorical data and the Mann–Whitney *U* test for continuous variables. The primary or secondary effectiveness endpoint was evaluated by χ^2^ test or Fisher’s exact test. Logistic regression analysis was used to estimate the effectiveness of PPSV23 in preventing pneumococcal pneumonia, nonpneumococcal pneumonia, and all-cause pneumonia. Kaplan–Meier methods were used to calculate the survival curves. The log-rank test was used for time to event analyses and Cox regression models were used to calculate hazard ratios. *P* values are two-tailed, with *P* < 0.05 considered statistically significant. All data processing and analyses were performed using the Statistical Analysis System (SAS) and SPSS version 18 software (SPSS, Chicago, IL, USA). According to the sample size calculation, 1600 evaluable patients were required (800 in each group). We assumed that the risk of pneumonia among RA patients was 2 cases per 100 patient-years and vaccine efficacy was 50% according to previous clinical studies and epidemiology data available in Japan (references). Also, a type I error probability of 0.05 (two-sided) and a statistical power of 80% were selected.

## Results

**Fig. 1:**
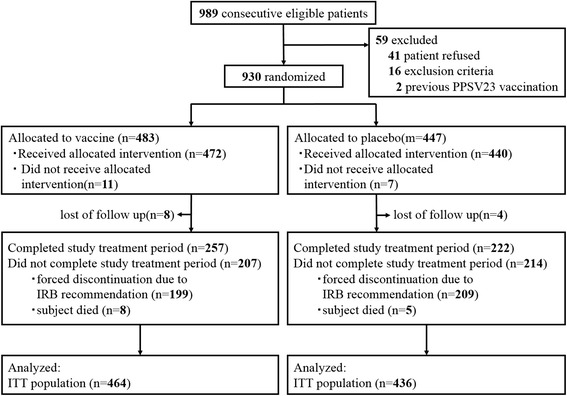
Flow diagram of patient enrollment. *IRB* institutional review board, *ITT* intention-to-treat, *PPSV23* 23-valent pneumococcal polysaccharide vaccine

Consecutive eligible patients between December 2012 and March 2014 were reviewed for inclusion. Of 989 patients, 59 patients were excluded (Fig. 1). The reasons for exclusion included inability to obtain the informed consent (*n* = 41) and conflict with the exclusion criteria (*n* = 18), including previous PPSV23 vaccination (*n* = 2). Among the remaining 930 patients, 18 patients withdrew consent after randomization. Thus, 912 patients were ultimately included; 472 were randomly assigned vaccine and 440 placebo.

The trial was conducted in accordance with the original protocol and there was no change in the outcome measures. In November 2014, however, the Committee on Immunization Practices of the Ministry of Health, Labour and Welfare in Japan stated that all adults ≥65 years of age should receive PPSV23 and began promoting routine vaccination with PPSV23. In response to this public comment, the NHO central IRB recommended stopping this trial continuation and vaccination with PPSV23 for all subjects receiving placebo after being keyed “open”. We decided trial discontinuation before the interim analysis according to this recommendation. Participants aged ≥65 years receiving placebo were thus assigned to receive PPSV23 vaccination and forced into discontinuation of follow-up until December 31, 2014.

In this study, participants in the forced discontinuation groups were keyed “open” and received PPSV23 even when they were assigned to receive placebo (Fig. 1). The primary endpoint was assessed in the ITT population, which included all randomized patients regardless of the forced discontinuation. Of 912 patients who were randomized to PPSV23 (*n* = 483) or placebo (*n* = 447), 12 patients lost to follow-up were excluded from the ITT analysis. The ITT population therefore included 900 patients (Fig. 1).

Demographic data for the two groups are presented in Table 1. The exposure time was 765.7 person-years (mean 1.7 years) in the vaccine group and 703.7 (mean 1.6 years) person-years in the placebo group. Patient characteristics were similar in both groups. During the follow-up period, pneumonia was diagnosed in 32 (3.6%) of the 900 patients; pneumonia was diagnosed in 15 (3.4%) of the 436 patients in the placebo group and in 17 (3.7%) of the 464 patients in the vaccine group. The incidence of pneumonia per 1000 person-years was 21.3 and 22.2 in the placebo and vaccine groups, respectively. The incidence rate for pneumonia in the vaccine group did not differ from that in the placebo group (*P* = 0.856).

**Table 1:** Baseline characteristics of 900 rheumatoid arthritis patients at randomization to PPSV23 or placebo

	Vaccine group	Placebo group	
	(*n* = 464)	(*n* = 436)	*P* value
Demographics			
Age (years)	63.3 ± 12.1	62.7 ± 11.8	0.529
Gender, male/female	90 (19.4%)/374 (80.6%)	106 (24.3%)/330 (75.7%)	0.074
Smoking history	90 (19.4%)	116 (26.6%)	0.010
Laboratory data			
CRP (mg/dl)	0.49 ± 1.11	0.51 ± 0.99	0.298
Serum albumin (g/dl)	4.06 ± 0.38	4.06 ± 0.37	0.926
Serum creatinine (mg/dl)	0.68 ± 0.36	0.68 ± 0.22	0.934
RA characteristics			
RA duration (years)	12.1 ± 10.4	11.6 ± 9.7	0.747
HAQ	0.66 ± 0.74	0.67 ± 0.75	0.918
DAS28 (CRP)	2.43 ± 1.10	2.51 ± 1.15	0.324
SDAI	8.00 ± 7.72	8.73 ± 8.55	0.252
CDAI	7.50 ± 7.38	8.24 ± 8.33	0.257
Comorbidity			
Cardiovascular disease	127 (27.4%)	131 (30.0%)	0.375
CVA	14 (3.0%)	10 (2.3%)	0.501
Ischemic heart disease	9 (1.9%)	9 (2.1%)	0.894
Hypertension	109 (23.5%)	116 (26.6%)	0.281
Arrythmia	6 (1.3%)	5 (1.1%)	0.842
Cardiac failure	6 (1.3%)	4 (0.9%)	0.415
Metabolic disease	111 (23.9%)	96 (22.0%)	0.498
Hyperlipidemia	72 (15.5%)	56 (12.8%)	0.251
Hyperuricemia	0	9 (2.1%)	0.001
Diabetes	48 (10.3%)	45 (10.3%)	0.991
CKD	17 (3.7%)	18 (4.1%)	0.719
Autoimmune disease	23 (5.0%)	14 (3.2%)	0.187
Rheumatoid lung	81 (17.5%)	71 (16.3%)	0.890
Interstitial pneumonia	56 (12.1%)	46 (10.6%)	0.473
Bronchial lesion	19 (4.1%)	19 (4.4%)	0.845
Pleural lesion	6 (1.3%)	6 (1.4%)	0.914
COPD	11 (2.4%)	16 (3.7%)	0.254
NTM	7 (1.5%)	8 (1.8%)	0.702
Treatment			
PSL	242 (52.2%)	214 (49.1%)	0.357
Dose of PSL (mg/day)	4.54 ± 2.79	4.71 ± 2.84	0.530
PSL ≥ 5 mg/day	130 (28.0%)	117 (26.8%)	0.691
MTX	300 (64.7%)	304 (69.7%)	0.106
Dose of MTX (mg/week)	8.01 ± 2.75	8.33 ± 2.85	0.227
MTX alone	40 (8.6%)	47 (10.8%)	0.273
MTX + PSL	54 (11.6%)	49 (11.2%)	0.851
MTX + biologics	103 (22.2%)	107 (24.5%)	0.406
TAC	60 (12.9%)	60 (13.8%)	0.714
Biologics	257 (55.4%)	253 (58.0%)	0.425

Data presented as number (percentage) or mean ± standard deviation

*PPSV23* 23-valent pneumococcal polysaccharide vaccine, *CRP* C-reactive protein, *RA* rheumatoid arthritis, *HAQ* Health Assessment Questionnaire Disability Index score, *DAS28* Disease Activity Score 28, *SDAI* simplified disease activity index, *CDAI* clinical disease activity index, *CVA* cerebrovascular accident, *CKD* chronic kidney disease, *COPD* chronic obstructive pulmonary disease, *NTM* nontuberculous mycobacteria, *PSL* prednisolone, *MTX* methotrexate, *TAC* tacrolimus

Sputum cultures and blood cultures were obtained in 18 and 14 episodes of pneumonia and urine samples for pneumococcal antigen detection were obtained in 21 episodes of pneumonia. An etiological diagnosis was obtained in 10 (15.6%) of the 64 episodes of pneumonia. Causative pathogens were identified in 12 (37.5%) of the 32 participants. Pneumococcal pneumonia was diagnosed in two participants in the vaccine group (2/17, 11.8%) and one participant in the placebo group (1/15, 6.7%). The causative agents of nonpneumococcal pneumonia were *Staphylococcus aureus* (*n* = 2), *Pseudomonas aeruginosa* (*n* = 2), *Klebsiella pneumoniae* (*n* = 1), *Haemophilus influenzae* (*n* = 1), *Escherichia coli* (*n* = 1), *Moraxella catarrhalis* (*n* = 1), and *Neisseria* sp. (*n* = 1). The incidence rate of pneumococcal pneumonia was not significantly different between the vaccine and placebo groups (Table 2). One adverse event (bronchial asthma) in the placebo group and three adverse events (interstitial pneumonia, mycoplasma pneumonia, heart failure) in the vaccine group were documented during the follow-up period.

**Table 2:** Incidence of primary endpoints in rheumatoid arthritis patients assigned to PPSV23 or placebo

	Vaccine group (*n* = 464)		Placebo group (*n* = 436)		
	Incidence rate per 1000 patient-years (*n*)	95% CI	Incidence rate per 1000 patient-years (*n*)	95% CI	*P* value
Pneumococcal pneumonia	2.6 (2)	0.7–9.5	1.4 (1)	0.3–8.0	0.523
Nonpneumococcal pneumonia	19.6 (15)	11.9–32.3	19.9 (14)	11.9–33.4	0.985
All-cause pneumonia	22.2 (17)	13.9–35.6	21.3 (15)	12.9–35.2	0.856

*PPSV23* 23-valent pneumococcal polysaccharide vaccine, *CI* confidence interval

**Fig. 2:**
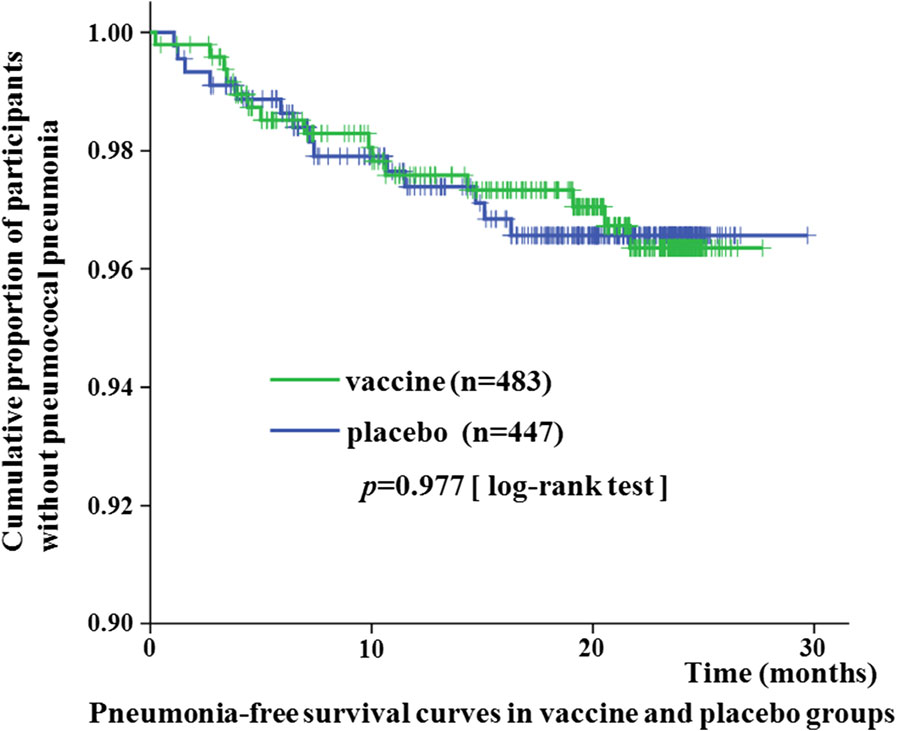
Pneumonia-free survival curves of RA patients receiving PPSV23 and placebo. Statistically significant difference was not observed between patients receiving PPSV23 and placebo (*P* = 0.084, log-rank test)

Kaplan–Meier survival curves were plotted for the pneumonia-free survival between the vaccine and placebo groups (Fig. 2). The pneumonia free-survival rates were not significantly different between the vaccine and placebo groups.

For stratified analyses based on risk for developing respiratory infections, patients were divided into the following groups: patients with rheumatoid lung disease, patients receiving biologics, patients receiving immunosuppressants (tacrolimus, mizoribine, and cyclosporine A), patients receiving more than 5 mg/day of prednisone, and patients classified as Steinbrocker stage 3 or 4. However, there was no significant difference in the number of pneumonia cases between the vaccine and placebo groups in each subgroup (Table 3). Also, as we planned, stratified analyses based on risk for developing respiratory infections were conducted according to the following groups: patients with rheumatoid lung disease, patients with RA treated with biological agents, patients with RA treated with immunosuppressive agents, patients receiving more than 5 mg/day of prednisone, and patients classified as Steinbrocker stage 3 or 4. However, there was no significant difference in the number of pneumonia cases between the vaccine and placebo groups in each subgroup (Table 3). Age categories were not considered because most of the study population were 65 years or older because of eligibility for pneumococcal vaccines in Japan.

**Table 3:** Primary endpoint (pneumonia) in each subgroup

Subgroup	PPSV23	Placebo	*P* value
With rheumatoid lung	12.3% (10/81)	5.6% (4/71)	0.1534
Receiving biologics	3.1% (8/257)	2.4% (6/253)	0.6085
Receiving immunosuppressants	4.1% (3/74)	2.9% (2/70)	0.6950
Receiving steroid (>5 mg/day of prednisolone)	3.9% (5/130)	2.6% (3/117)	0.5698
With advanced stage	3.7% (9/246)	3.8% (9/239)	0.9502

Data presented as percentage (number)

*PPSV23* 23-valent pneumococcal polysaccharide vaccine

**Fig. 3:**
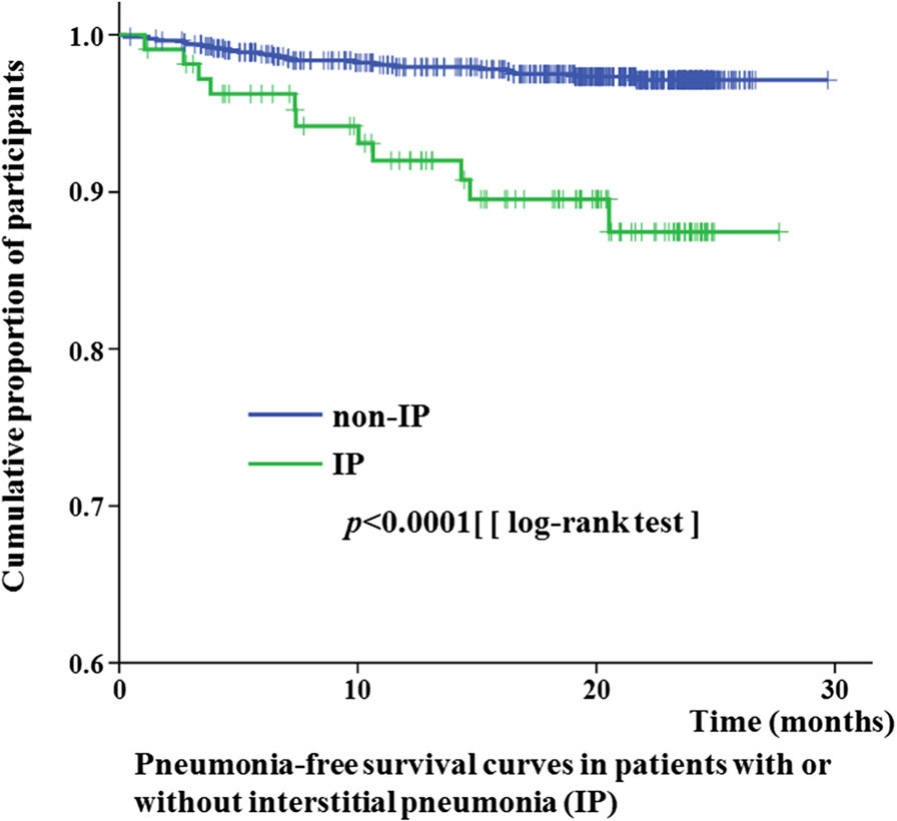
Pneumonia-free survival curves of RA patients with or without interstitial lung disease (ILD). Curves are stratified by the presence or absence of ILD. Statistically significant differences were observed between patients with or without ILD (*P* < 0.0001, log-rank test)

Finally, we compared baseline data between patients with or without pneumonia in the total subjects. A few of the demographic and clinical variables were associated with risk of pneumonia, as shown in Table 4. Older age and the presence of interstitial pneumonia were found to be predictive factors for pneumonia. The effect of treatment variables on the risk of pneumonia was analyzed using univariate analyses (Table 5); however, no treatment variable was identified as a risk factor for pneumonia. A multivariate analysis of all variables is presented in Table 6. The presence of interstitial pneumonia remained a significant risk factor for pneumonia in RA patients. Kaplan–Meier curves stratified by the presence of interstitial pneumonia showed a significant difference between RA patients with or without interstitial pneumonia. Additionally, the presence of interstitial pneumonia significantly affected the pneumonia-free survival rates of RA patients (Fig. 3).

**Table 4:** Baseline clinical and demographic data between RA patients with or without pneumonia

	Pneumonia	Without pneumonia	
	(*n* = 32)	(*n* = 868)	*P* value
Demographics			
Age (years)	68.6 ± 10.8	62.8 ± 11.9	0.010
Gender, male/female	7 (21.9%)/25 (78.1%)	189 (21.8%)/679 (78.2%)	0.989
Smoking history	7 (21.9%)	199 (22.9%)	0.889
Laboratory data			
CRP (mg/dl)	1.14 ± 2.29	0.48 ± 0.97	0.023
Serum albumin (g/dl)	3.95 ± 0.44	4.07 ± 0.37	0.210
Serum creatinine (mg/dl)	0.70 ± 0.21	0.68 ± 0.31	0.248
RA characteristics			
RA duration (years)	12.3 ± 8.3	11.9 ± 10.1	0.378
HAQ	0.78 ± 0.81	0.66 ± 0.74	0.564
DAS28 (CRP)	2.32 ± 0.97	2.48 ± 1.13	0.548
SDAI	6.72 ± 6.11	8.41 ± 8.20	0.237
CDAI	5.64 ± 4.76	7.94 ± 7.94	0.123
Comorbidity			
Cardiovascular disease	13 (40.6%)	245 (28.2%)	0.128
CVA	2 (6.3%)	22 (2.5%)	0.209
Ischemic heart disease	1 (3.1%)	17 (2.0%)	0.482
Hypertension	10 (31.3%)	215 (24.8%)	0.406
Arrythmia	2 (6.3%)	9 (1.0%)	0.055
Cardiac failure	2 (6.3%)	8 (0.9%)	0.046
Metabolic disease	10 (31.3%)	197 (22.7%)	0.259
Hyperlipidemia	6 (18.8%)	122 (14.1%)	0.297
Hyperuricemia	0	9 (1.0%)	0.721
Diabetes	5 (15.6%)	88 (10.1%)	0.229
CKD	2 (6.3%)	33 (3.8%)	0.356
Autoimmune disease	1 (3.1%)	36 (4.1%)	0.617
Rheumatoid lung	14 (43.8%)	138 (15.9%)	<0.0001
Interstitial pneumonia	11 (34.4%)	91 (10.5%)	<0.0001
Bronchial lesion	3 (9.4%)	35 (4.0%)	0.148
Pleural lesion	1 (3.1%)	11 (1.3%)	0.354
COPD	0	27 (3.1%)	0.371
NTM	1 (3.1%)	14 (1.6%)	0.422
PPSV23 vaccination	17 (53.1%)	447 (51.5%)	0.856
Treatment			
PSL	18 (56.3%)	438 (50.5%)	0.520
Dose of prednisolone (mg/day)	5.61 ± 4.90	4.58 ± 2.69	0.521
MTX	20 (62.5%)	584 (67.3%)	0.572
Dose of MTX (mg/week)	7.90 ± 2.38	8.18 ± 2.82	0.660
TAC	4 (12.5%)	116 (13.4%)	0.573
Biologics	14 (43.8%)	496 (57.1%)	0.133

Data presented as number (percentage) or mean ± standard deviation

*PPSV23* 23-valent pneumococcal polysaccharide vaccine, *CRP* C-reactive protein, *RA* rheumatoid arthritis, *HAQ* Health Assessment Questionnaire Disability Index score, *DAS28* Disease Activity Score 28, *SDAI* simplified disease activity index, *CDAI* clinical disease activity index, *CVA* cerebrovascular accident, *CKD* chronic kidney disease, *COPD* chronic obstructive pulmonary disease, *NTM* nontuberculous mycobacteria, *PSL* prednisolone, *MTX* methotrexate, *TAC* tacrolimus

**Table 5:** Predictors of pneumonia in RA patients by the Cox-hazard model (univariate analysis)

	Hazard ratio	*P* value	95% CI
Demographics			
Age (years)	1.058	0.002	1.021–1.095
Gender, male	1.073	0.870	0.464–2.480
Laboratory data			
CRP (mg/dl)	1.321	<0.0001	1.140–1.531
Serum albumin (g/dl)	0.414	0.051	0.171–1.004
Serum creatinine (mg/dl)	1.358	0.413	0.652–2.828
RA characteristics			
RA duration (years)	1.002	0.927	0.968–1.036
HAQ	1.219	0.366	0.794–1.871
DAS28 (CRP)	0.894	0.498	0.646–1.237
SDAI	0.970	0.281	0.919–1.025
CDAI	0.949	0.115	0.890–1.013
Comorbidity			
Smoking history	0.936	0.877	0.405–2.165
Cardiovascular disease	1.745	0.122	0.862–3.533
Metabolic disease	1.612	0.211	0.763–3.405
CKD	1.814	0.415	0.433–7.592
Autoimmune disease	0.723	0.750	0.099–5.299
Interstitial pneumonia	4.449	<0.0001	2.143–9.237
PPSV23 vaccination	1.048	0.894	0.523–2.099
Treatment			
PSL	1.314	0.443	0.654–2.643
Dose of prednisolone (mg/day)	1.117	0.102	0.978–1.276
MTX	0.778	0.493	0.381–1.593
Dose of MTX (mg/week)	0.962	0.634	0.821–1.128
TAC	0.956	0.934	0.335–2.727
Biologics	0.544	0.088	0.270–1.094

*PPSV23* 23-valent pneumococcal polysaccharide vaccine, *CRP* C-reactive protein, *RA* rheumatoid arthritis, *HAQ* Health Assessment Questionnaire Disability Index score, *DAS28* Disease Activity Score 28, *SDAI* simplified disease activity index, *CDAI* clinical disease activity index, *CKD* chronic kidney disease, *PSL* prednisolone, *MTX* methotrexate, *TAC* tacrolimus, *CI* confidence interval

**Table 6:** Predictors of pneumonia in RA patients by the Cox-hazard model (Multivariate analysis)

	Hazard ratio	*P* value	95% CI
Demographics			
Age (years)	1.039	0.068	0.997–1.083
Gender, male	0.580	0.317	0.200–1.686
Smoking history	1.247	0.670	0.452–3.443
Laboratory data			
CRP (mg/dl)	1.384	0.268	0.779–2.459
Serum albumin (g/dl)	0.917	0.877	0.305–2.758
Serum creatinine (mg/dl)	1.550	0.366	0.599–4.005
RA characteristics			
RA duration (years)	1.010	0.582	0.974–1.048
HAQ	1.202	0.506	0.699–2.067
DAS28 (CRP)	1.026	0.936	0.545–1.933
SDAI	0.945	0.845	0.534–1.670
CDAI	0.944	0.851	0.517–1.723
Comorbidity			
Cardiovascular disease	1.138	0.749	0.515–2.518
Metabolic disease	1.283	0.542	0.576–2.856
CKD	1.402	0.689	0.268–7.344
Autoimmune disease	0.468	0.494	0.053–4.137
Interstitial pneumonia	3.611	0.003	1.553–8.399
PPSV23 vaccination	0.849	0.656	0.414–1.743
Treatment			
PSL	1.081	0.846	0.492–2.375
MTX	1.431	0.390	0.632–3.240
TAC	0.712	0.552	0.232–2.182
Biologics	0.664	0.307	0.302–1.458

*PPSV23* 23-valent pneumococcal polysaccharide vaccine, *CRP* C-reactive protein, *RA* rheumatoid arthritis, *HAQ* Health Assessment Questionnaire Disability Index score, *DAS28* Disease Activity Score 28, *SDAI* simplified disease activity index, *CDAI* clinical disease activity index, *CKD* chronic kidney disease, *PSL* prednisolone, *MTX* methotrexate, *TAC* tacrolimus, *CI* confidence interval

## Discussion

The effectiveness of PPSV23 in preventing pneumococcal pneumonia remains controversial, especially in high-risk individuals [14]. Although several studies have demonstrated the effectiveness of PPSV23 [15], few studies have focused on patients with autoimmune diseases. To our knowledge, this is the first RCT to evaluate this concern in patients with RA. We demonstrated previously that PPSV23 vaccination induced the serotype-specific IgG and functional opsonization index responses were not impaired even in RA patients receiving immunosuppressive treatments [11]. However, we were unable to show that PPSV23 was effective in the prevention of pneumonia overall or pneumococcal pneumonia. This might be because of lack of power and shortened follow-up periods. The fundamental difficulty in obtaining a definitive pathological cause of pneumonia may also be related to these negative data. Definitive pneumococcal pneumonia (positive blood or sputum culture) is the most specific outcome to evaluate vaccine effectiveness; however, it has a low sensitivity. Therefore, we used the BinaxNOW® *S. pneumoniae* Antigen Card test for diagnosis [16]. However, 13 of 34 pneumonia cases did not receive this urinary antigen test and only a fraction of pneumonia cases caused by *S. pneumoniae* were included in our analysis. A Cochrane review also failed to show any protective efficacy of PPSV23 in patients with chronic pulmonary diseases [17]. Further high-quality trials with sufficient sample size would be useful to confirm our findings. Pneumococcal conjugate vaccines offer an alternative approach to prevent pneumococcal disease. The seven-valent pneumococcal conjugate has been shown to protect HIV-infected adults from recurrent pneumococcal infection [18]. The effectiveness of PPSV23 preventing pneumococcal infections among patients with chronic pulmonary diseases is unclear [19]. However, the efficacy and cost-effectiveness of PPSV23 in preventing IPD among the general population had been demonstrated [20]. Based on the new guidelines, the sequential administration of conjugate and polysaccharide pneumococcal vaccines has recently been the most appropriate approach for the prevention of pneumonia in the general population [21]. Our study confirmed that polysaccharide vaccine alone is not effective for prevention of pneumonia. Therefore, sequential administration of PCV13 and PPSV23 could also be an appropriate approach for the prevention for pneumonia in RA patients receiving immunosuppresssive treatments.

Pneumonia is the most common type of infection in RA [22]. Previous studies demonstrated increased frequencies of pneumonia with higher mortality in RA patients [23]. Our data showed that the incidence of pneumonia was 2.14 per 100 person-years in RA patients with a relatively high risk for infections. Glucocorticoid use was also shown to increase the risk of serious infections in a dose-dependent manner in RA patients [24]. The magnitude of the risk of infection associated with prednisolone dose > 10 mg/day was similar to that associated with TNF-α antagonists [25]. However, neither glucocorticoid dosage nor bDMARD use was demonstrated to be a predictor of pneumonia risk in our study. Of note, we found no evidence for increased rates of pneumonia associated with bDMARD use. However, our data are inconsistent with a recent meta-analysis of RCTs of RA patients who received bDMARDs [26]. Nevertheless, the included patients and analytical methods in previous studies differed significantly from those in this study. Additionally, the relatively lower corticosteroid dosage prescribed to our RA patients may have influenced the association between glucocorticoid dosage and the occurrence of pneumonia.

As reported for RA patients, older age and pulmonary disease were found to be independently associated with bacterial infection. Among variables, preexisting interstitial pneumonia and older age were demonstrated to be associated with the risk of pneumonia [27]. Among the extra-articular manifestations of RA, there has been renewed interest in pulmonary complications directly associated with RA, which manifest as a variety of clinical signs such as pleural disease, pulmonary nodules, interstitial lung disease (ILD), and airway disease [28]. A large observational cohort study in Japan showed that the presence of ILD is one of the factors associated with increased mortality in RA patients [29]. Our data are consistent with these previous studies. Although the pathophysiologic mechanism for this finding has not been elucidated, decreased ciliary and respiratory epithelial function seen in patients with interstitial pneumonia may contribute to the higher frequency of pneumonia [30].

There are several limitations associated with this study. First, the forced discontinuation of follow-up is unusual and the disconfirmation rates were high. Thus, potential bias introduced by the discontinuation group (≥65 years of age) should be considered. Second, our sample size was limited. For sample size calculation, we assumed, from previously published data on RA patients under biologic treatment [31], a rate of pneumonia of 2.5/100 patient-years. Based on these assumptions, we primarily calculated that we needed 714 patients receiving PPSV23 and 714 patients receiving placebo to confirm 50% reduction of pneumonia occurrence during 2 years of follow-up at an α error of 0.10 and a β error of 0.80. However, we could not reserve sufficient sample size, and the possibility of an underpowered study cannot be denied. Third, our RA population consisted of groups at high risk for infections, and thus our results may not be generalizable to other RA populations. Fourth, we cannot rule out the imprecision of our complete data due to unreliability in the diagnosis of pneumonia.

The strengths of our study include examination of an RA patient cohort using data captured from the largest NHO data center. Laboratory results and physician notes are available in this data resource and allow for examination of potential risk factors that are not typically available when using only administrative claims data. Our study represents the most comprehensive, hospital-based study with the outcomes in all enrolled patients followed completely. The participants in this study, in contrast to those in many clinical trials, were similar demographically to general RA patients. Our data can therefore provide a great deal of useful information about generalizability.

## Conclusions

Our data showed that PPSV23 vaccination was not effective in preventing pneumonia. While PPSV23 vaccination is recommended for adults ≥65 years of age, our results suggested uncertainty regarding its effectiveness for pneumonia in RA patients at high risk for infections. Clinicians should keep in mind the patient’s age and the presence of interstitial pneumonia because such patients are at an increased risk of developing pneumonia.

## Abbreviations

ILD: Interstitial lung disease
NHO: Japanese National Hospital Organization
PCV: Pneumococcal conjugate vaccine
PPV23: 23-Valent pneumococcal polysaccharide vaccine
RA: Rheumatoid arthritis
